# Chemical Composition of Secretions from Facial Glands of Captive Hoary Bat (*Lasiurus cinereus*) in Virginia

**DOI:** 10.1007/s10886-025-01593-3

**Published:** 2025-03-20

**Authors:** Kristina R. Stefaniak, Lark Kellogg, Matthew T. Close, Karen E. Powers

**Affiliations:** 1 Chemistry Department, Radford University, Radford, VA 24142 USA; 2 Southwest Virginia Wildlife Center of Roanoke, Roanoke, VA 24018 USA; 3 Biology Department, Radford University, Radford, VA 24142 USA

**Keywords:** Facial Skin glands, Sudoriferous gland, Sebaceous gland, *Lasiurus cinereus*, Hoary bat, Chemical communication

## Abstract

Our study represents the first known report of hoary bat (*Lasiurus cinereus*) facial gland histology and chemical characterization of facial gland secretions. While in captivity from October 2023 to February 2024, facial secretions of a male hoary bat were observed and collected. Gas chromatography-mass spectrometry (GC-MS) analysis of a methanol extraction of November 2023 samples identified 23 compounds after comparing retention times, mass spectra, and Kovats retention index. These compounds consisted of alkanes, alcohols, ketones, terpenoids, monoacylglycerols, and androgens. Of interest was the identification of five androstane derivatives, testosterone metabolites, that have not been seen previously in mammal secretions and indicate possible mating readiness. The chemical analyses from November swabs and histological examination of facial skin in February suggest that this male was actively secreting mate-signaling compounds throughout late fall and winter months. Anecdotal mating records on wintering grounds combined with this bat’s warm captive environment likely allowed for this extended mating readiness for his many months at the wildlife center.

## Introduction

On 19 July 2023, an adult male hoary bat (*Lasiurus cinereus*) was admitted to the Southwest Virginia Wildlife Center of Roanoke with a fractured right humerus. It was brought into care from a break sustained at the Clinch Mountain Wildlife Management Area in Cedar Bluff, VA. At intake, the hoary bat weighed 22.6 g and had a right forearm length of 55.1 mm. Two days after intake, the bat’s humerus was pinned by veterinarian Dr. K. Thomasson.

**Fig. 1:**
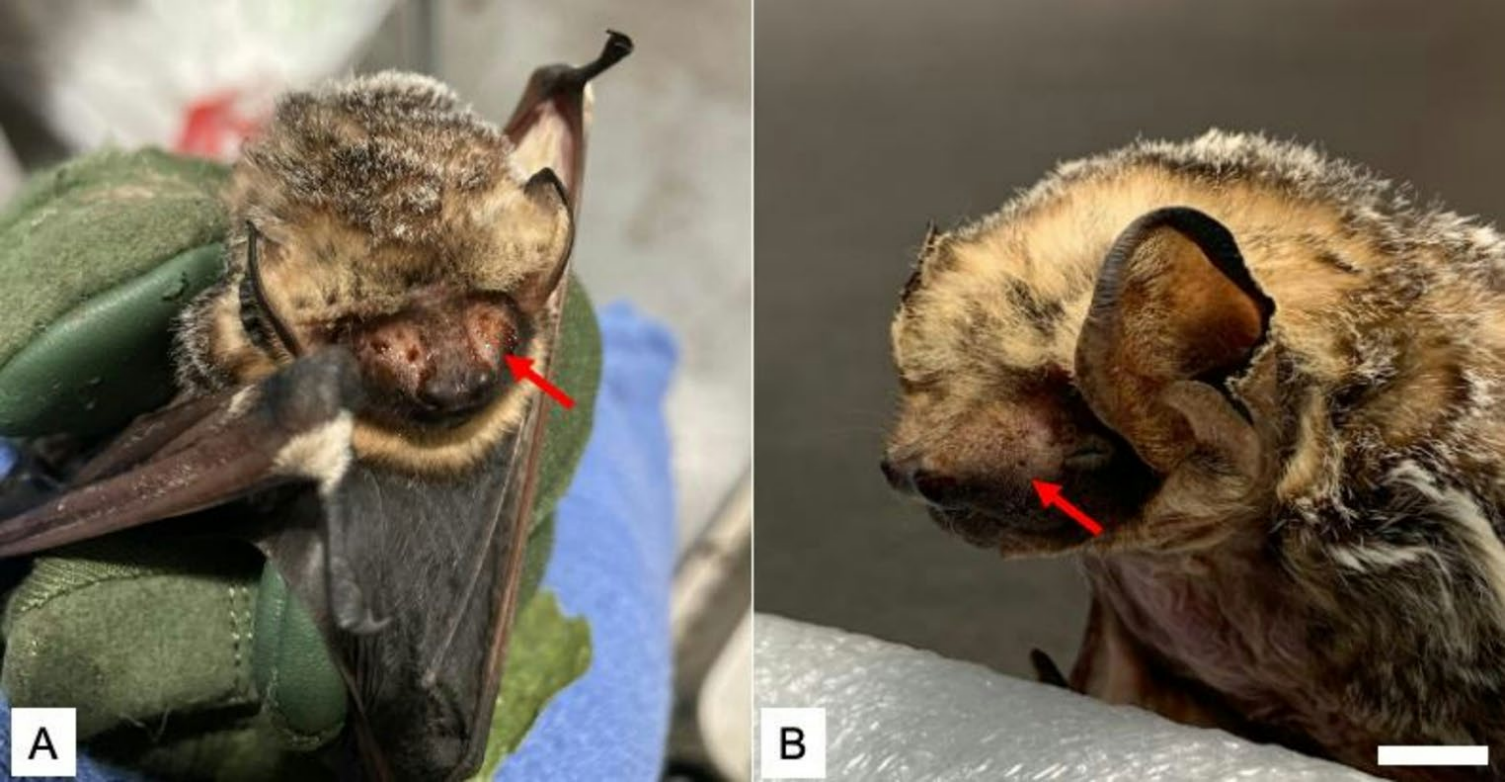
Captive adult male hoary bat (*Lasiurus cinereus*) facial glands. **A**. Live bat at the Southwest Virginia Wildlife Center of Roanoke (Virginia) with visible facial gland excretions on 28 October 2023. **B**. The same bat following euthanasia and immediately prior to tissue collection and fixation on 14 February 2024. Scale bar = 5 mm. Red arrows indicate locations of facial glands and the source of secretion and tissue collection for this study Photo (**a**) taken by LK, photo (**b**) taken by MTC

Over the next four months in care, the bat required several subsequent surgeries: pin removal (performed by Dr. E. Dominguez) and two minor amputations near the third knuckle of the second phalange of the right wing due to idiopathic inflammation of the knuckle joint (performed by Dr. K. Thomasson). We first observed secretions from the facial glands on 28 October 2023, 7 d after the removal of the pin (Fig. 1). The sebaceous secretions continued to occur with no discernable pattern throughout November, and then ceased until secretions resumed in January. The bat’s health slowly declined in captivity and he was ultimately euthanized on 14 February 2024.

The morphology of bat facial glands has been described for the genus *Lasiurus* by Werner and Dalquest (1952). Facial glands include salivary glands (sublingual and submaxillary), sweat or sudoriferous glands around the jaws, and sebaceous glands (often associated with hair follicles). Within this genus, there are no known differences in glandular make-up between sexes (Dalquest and Werner 1954). One member of Family Vespertilionidae, the black-winged little yellow bat (*Rhogeessa tumida*), expresses sexual dimorphism in scent glands; males have them at the base of their ears, while they are absent in females (Scully 1977). Gland placement is thought to direct its utility. For example, waxy secretions from shoulder glands in male northern yellow-shouldered-bats (*Sturnira parvidens*) are thought to influence conspecific recognition, reproduction (the odor may increase female attraction), and possibly competition (male-male communication; Faulkes et al. 2019). Male greater sac-winged bats (*Saccopteryx bilineata*) store a mixture of glandular oils, urine, and saliva in their wing-sacs, though the storage sacs are not glandular in origin (Voigt and Von Helversen 1999). This composite is expressed from the males’ wing sacs in hovering displays around multiple females, with the ultimate goal of increasing the males’ harem size (Voigt and Von Helversen 1999). The chest glands of male spear-nosed bats (*Phyllostomus hastatus*) produce different chemical secretions which communicate mating status and individual identity (Adams and Wilkinson 2018).

Neither has the function of the facial gland secretions in the genus *Lasiurus* been described, nor has its chemical composition been examined. To that point, we sought to determine the make-up of our captive hoary bat’s facial secretions. The composition might provide clues as to the functionality of the oils, whether they are a response to stress in captivity or a seasonal effect of fall or winter bat reproductive efforts. Despite the small sample size, our ability to collect multiple samples from a captive bat over an extended period provided the opportunity to examine the temporally associated secretions across multiple samples, searching across a variety of chemical classes of molecules, and fully exploring our findings using gas chromatography–mass spectrometry (GC-MS) technology. In addition, facial gland structure was determined through histology and allowed for comparison between chemical composition and possible social behavior.

## Methods

### Sample Collection and Preservation

We opportunistically collected six swab samples from the live hoary bat from 11 November 2023 until 29 November 2023. Each sample was obtained by gently dabbing a sterile foam swab against the rostrum of the bat until all liquid had been absorbed. The foam swabs were then placed into 0.7 mL Eppendorf tubes. The first collection on 11 November 2023, sample A, was submerged in 0.3 mL of 70% ethanol, while the rest were stored without a liquid medium and kept at 4 °C, subsequently labeled samples B-F.

### Histology

Following euthanasia, the carcass was kept on ice and transported from the Southwest Virginia Wildlife Center to Radford University. Within 5 h, a region from the left side of the bat’s face that was previously swabbed and presumed to contain facial glands (Fig. 1) was dissected from the bat’s head and fixed in 10% neutral buffered formalin. The remaining carcass was prepared as a museum specimen, and the voucher is now housed in the Biology Department at Radford University (RU 18332).

Skin samples were dehydrated through a series of graded ethanols and infiltrated and embedded in Paraplast embedding medium (Leica Biosystems Inc., Chicago, IL). The tissue was sectioned into 10-µm thick serial sections using a Leica RM2125RTS microtome (Leica Biosystems Inc., Chicago, IL). Sections were mounted to glass microscope slides in two staining series, and slides were stained with either Hematoxylin and Eosin for general nuclear and cytoplasmic staining or modified Masson’s Trichrome stain to differentiate connective tissues, muscle and epithelial tissues. Slides were examined and photographed using an Accu-Scope EXC-500 Compound Light Microscope fitted with an Excelis HD Camera (Accu-Scope Inc., Commack, NY). Glands were then characterized based on their structure, composition and staining properties.

### GC-MS Analysis

Samples were prepared by adding 500 µL methanol (J.T. Baker, LC-MS grade) to each swab, vortexed to mix, and placed in a water bath sonicator at room temperature for 10 min. The methanol plus extracted compounds were transferred to gas chromatography vials. Blank extractions were performed using sterile swabs following the same extraction protocol.

The samples were analyzed on a Shimadzu GC-2010 Plus with TQ8040 mass spectrometer using a SH-Rxi-5Sil MS column (30 m and 0.25 ID). The carrier gas was helium at a flow rate of 7.8 mL/min. Analysis was performed using a 1 µL splitless injection at 250 °C. The starting column temperature was 60 °C with a 5 °C/min ramp to 280 °C, held for 1 min, then a 30 °C/min ramp to 300 °C and held for 3 min. The ion source and detector interface were set to 250 °C and a Q3 scan from 40 to 600 m/z was performed.

### Peak Identification

All chromatography spectra were analyzed in Shimadzu GCMS Postrun software. After baseline correction the peaks were identified by comparing the mass spectral data with the National Institute for Standards and Technology (NIST) 2017 database. The compounds that appeared in the blank extractions were removed from the analysis. An in-house database was created from the retention time and NIST search results. From this database, compounds could be identified in subsequent samples. In addition, the ASTM D2887 Calibration Mix (Millipore-Sigma) was used to calculate Kovats retention indices for our instrument and method parameters. The Kovats retention index was calculated for each compound identified and compared with published values in NIST or Human Metabolome Database (HMDB).

## Results

### Histology

**Fig. 2:**
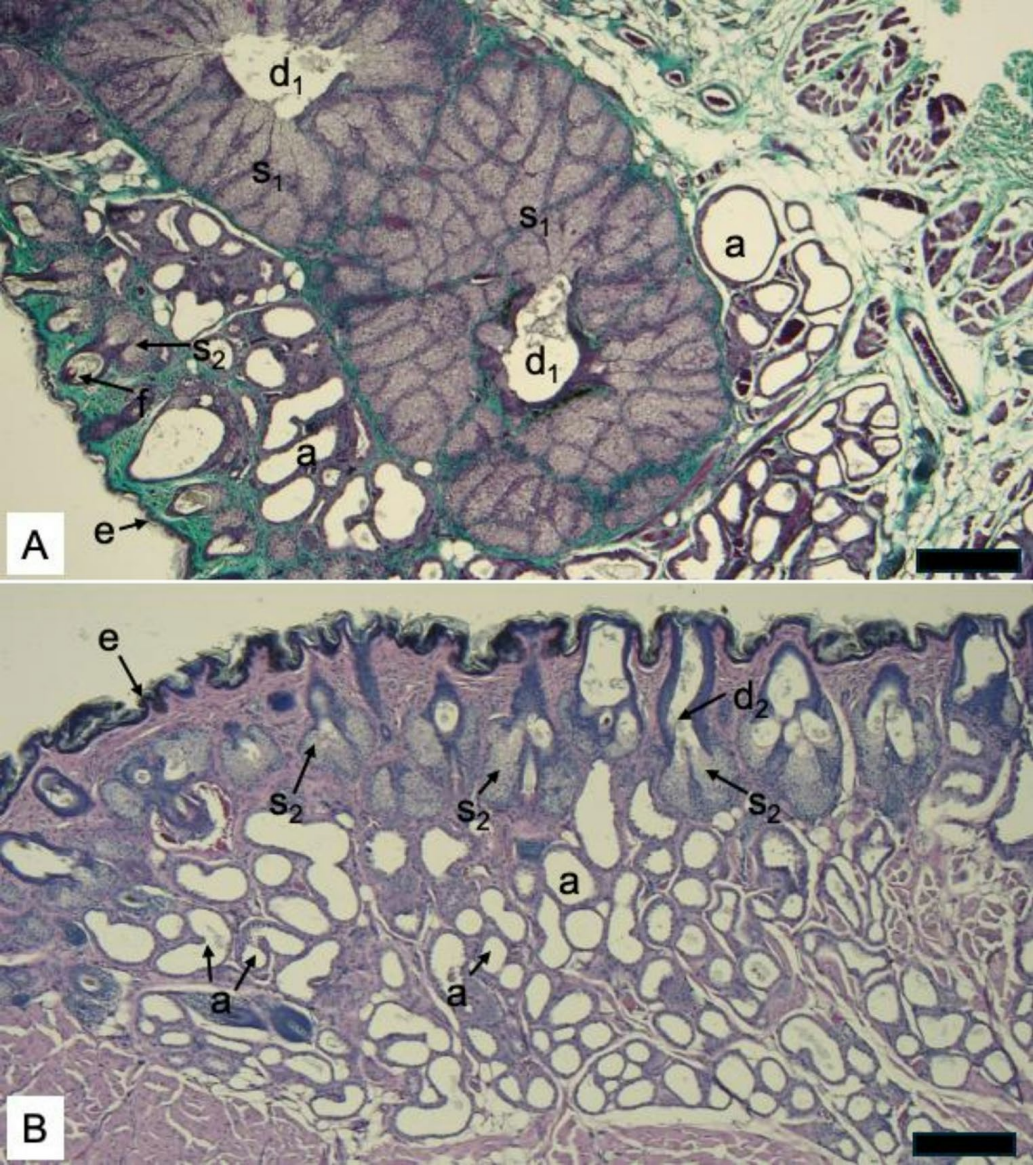
Distribution and diversity of sebaceous glands in captive male hoary bat (*Lasiurus cinereus*) facial skin. **A**. Image showing large, highly branched sebaceous glands (s_1_) that join a single central duct (d_1_) the deep dermal layer of skin. Smaller sebaceous glands (s_2_) are associated with the hair follicles (f) and are distributed more superficially in the dermis and immediately deep to the epidermis (e). Tubular sudoriferous glands (a) are also distributed around, but mainly superficial to, these large sebaceous glands in sections examined. Modified Masson’s trichrome stain. 100x magnification, Scale bar = 50 mm. **B**. Image showing distribution of small sebaceous glands (s_2_) and ducts (d_2_) associated with hair follicles. Sudoriferous glands (a) are distributed deep to the sebaceous glands (s_2_) and are in great abundance through most of the bat facial gland area. H&E stain. 100x magnification, Scale bar = 50 mm. Photo taken by MTC

**Fig. 3:**
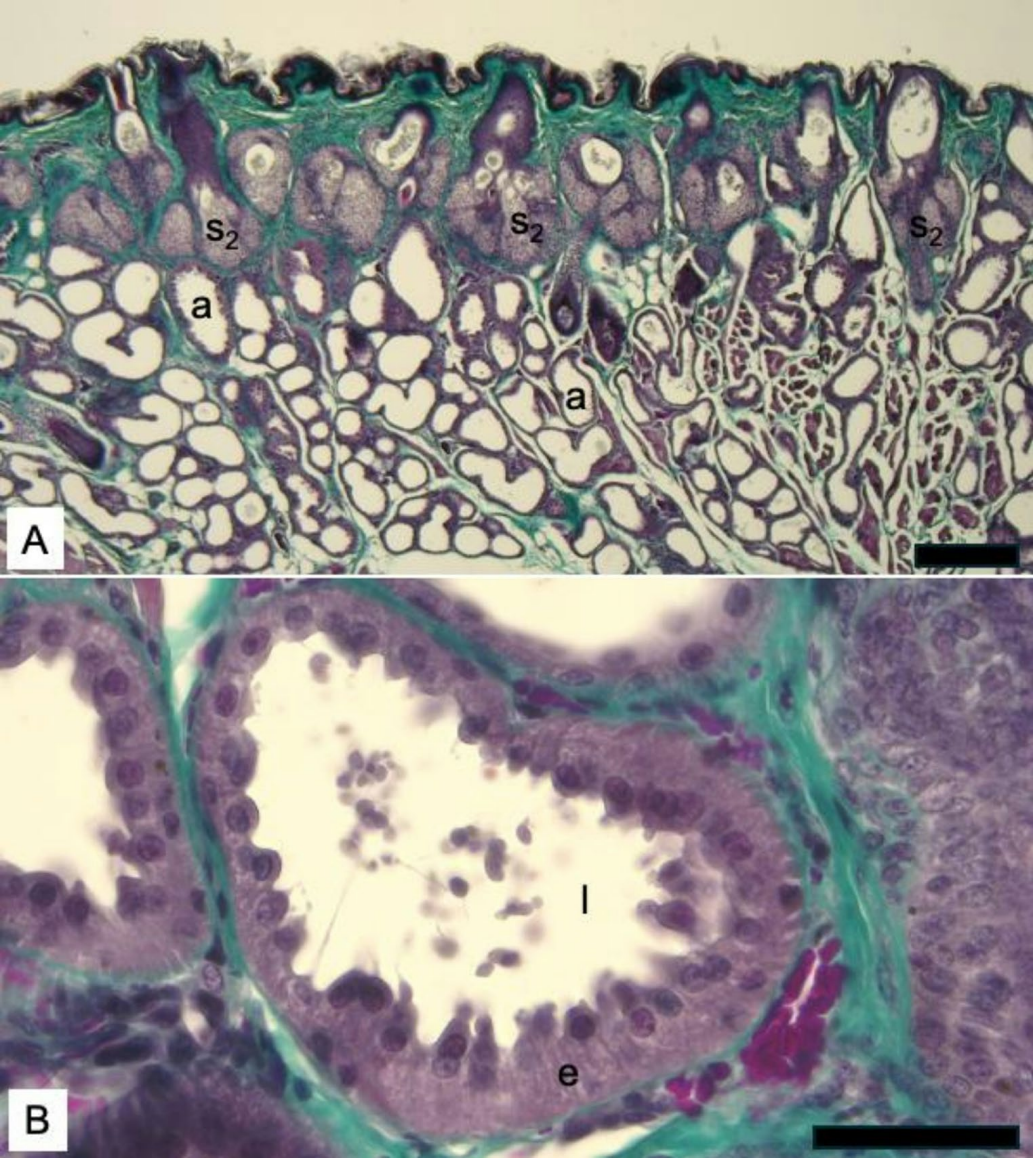
Distribution of sudoriferous glands in captive male hoary bat (*Lasiurus cinereus*) facial skin. **A**. Sudoriferous glands (a) are widely distributed in the dermis deep to the superficial sebaceous glands (s_2_). Trichrome stain. 100x magnification, Scale bar = 100 mm. **B**. High magnification image (600x) of a sudoriferous gland showing actively secreting cells. At the time the sample was fixed, the epithelial cells (e) were in active states of secretion, as evidenced by the sloughing off of the apices into the lumen (l) of the duct. Modified Masson’s trichrome stain. Scale bar = 50 mm. Photos taken by MTC

The region of skin sampled was composed of a relatively thin epidermis and a thick dermis that contained a high density of both sebaceous (Fig. 2) and sudoriferous glands (Fig. 3). The sebaceous glands were of two types: large, highly branched and lobed glands located deeper in the dermis that converged on a single large duct that eventually opened to the skin’s surface (Fig. 2a) and smaller, superficial sebaceous glands composed of less than 4 lobes and associated with and emptying into hair follicles (Fig. 2b). The larger sebaceous glands closely resembled the Meibomian glands, another type of sebaceous gland from the lower eyelids, as reported by Rehorek et al. (2010) for some bat species.

The tubular, sudoriferous glands were located throughout the dermis (Fig. 3a) and, when compared to the sebaceous glands, were the dominant gland type in the region sampled. Similar to previous studies in other bat species (Dalquest and Werner 1954; Rehorek et al. 2010), regions of the dermis where the sudoriferous glands were the main gland type were thicker than regions where large sebaceous glands were predominant. Based on light microscopy, the sudoriferous glands observed were of the apocrine type, where exocytosis occurs via “decapitation secretion” of blebs/snouts at the apical borders of the cells. These glands ranged from small in diameter and finely dilated to large in diameter and highly dilated in appearance (Fig. 3a). This variation in apparent diameter resulted, in part, from variation in thickness of the epithelium of the glands, where a thin epithelium presented with a large dilated lumen and a thick epithelium presented with a constricted lumen. The variation in thickness of the epithelium was underscored by the loss or shedding of the apical borders of these cells into the lumen of the ducts (Fig. 3b) as is the known type of secretion mechanism of this gland type. Higher magnification images of many sudoriferous glands showed extensive sloughing of cellular contents from cell apices into the lumen of the duct (Fig. 3b), indicating that these glands were actively secreting until the time of death. Our results therefore serve as a snapshot of the condition of the facial skin during this active secretory phase, with both dilated and actively secreting apocrine sudoriferous glands.

### Compounds Identified

The blank swab extraction consisted of 30 compounds including alkanes, plasticizers, and polyethylene glycol derivatives. If these peaks were seen in the oil secretion samples they were attributed to the extraction process and removed from the in-house database. After testing the six secretions our in-house database consisted of 57 compounds. Thirteen of these remain unidentified, as their fragmentation pattern did not match any in the NIST spectral database. This could be due to co-elution or possibly a chemical not contained in the database. The remaining 44 compounds were identified through the NIST spectral library and calculated Kovats retention indices (RI) were compared to reported values. Table 1 lists the 23 chemicals that matched both the NIST spectral database and literature RI values. Literature Kovats RI could not be found for 3 of the peaks but mass spectral fragmentation patterns showed a high similarity (Table 1). Additionally, the Kovats RI could not be calculated for the first peak, methyl isobutyl ketone, due to the eight hydrocarbon standard eluting with the solvent. However, the NIST mass spectral library match was 94% and the RI literature value of 730 agrees with the retention location of that peak. Undecane was triple confirmed by a matching retention time using an undecane standard.

Using the in-house database the frequency of the chemicals could also be determined. Seven chemicals only appeared in one of the collected secretions while two were found in all six collected samples. During the two-week collection window, the secretions exhibited a changing chemical composition. Although sample B exhibited many peaks on the chromatogram only two chemicals matched with the in-house database. Additionally, sample B included no androgen compounds which are seen in the remaining five samples (Table 1).

**Table 1:** Chemicals identified from six facial swabs from a captive male hoary Bat (*Lasiurus cinereus*) collected 11–29 November 2023. These chemicals were identified through NIST mass spectral data and experimental Kovats retention index. The chemical class and the frequency the chemical found in the six samples are included.

Chemical Name	Kovats RI	Chemical Family	Sample
Methyl isobutyl ketone	Not calculated	ketone	A
Undecane^a^	1100	alkane	A
Dodecanal	1404	aldehyde	A
2,6-Di-tert-butyl-4-hydroxy-4-methyl-2,5-cyclohexadien-1-one	1429	monocyclic monoterpenoids	A, C, D, E, F
2,6-Dihydroxybenzoic acid, 3TMS derivative	1434	salicylic acid TMS derivative	A
Dedecanol	1437	alcohol	D
3-Butenyl adipate^c^	1470	-	D, E, F
1,1’-[oxybis(methylene)]bis-cyclohexane	1678	-	C, D, E, F
2,4-Diphenyl-4-methyl-2(E)-pentene	1813	-	C, D, E
18-Norabieta-8,11,13-triene	1888	tricyclic	A, B, C, D, E, F
Dehydroabietal	2004	diterpenoid	A, B, C, D, E, F
Abietatriene	2011	diterpenoid	D, E
N-Methyldidecylamine^c^	2032	tertiary amine	A
1-Methyl-10,18-bisnorabieta-8,11,13-triene^c^	2043	diterpenoid	C, D, E, F
5-Androstene-3β,16β,17α-triol	2049	androgen	C, D, E
Androstan-3-one, 17-hydroxy-2-methyl-, (2β,5β,17β)-	2074	androgen	C, D, E
5β-Androstane-3β,17β-diol	2080	androgen	D, E
5β-Androstane	2092	androgen	A, C, D, E, F
5α-Androstane	2094	androgen	A, C, D, E, F
Kaurenoic acid^b^	2100	diterpenoid	D, E
2-Monopalmitin	2415	monoacylglycerol	A, C, E
Methyl dehydroabietate	2438	diterpenoid	A, D, E, F
1-Monostearin	2443	monoacylglycerol	D

^**a**^Retention time of peak matched standard

^b^Only retention index for methyl ester derivative found in literature

^c^Literature retention index could not be found

## Discussion

The glandular faces of bats have been previously investigated for both their unique morphological characteristics (Dalquest and Werner 1954) as well as the correlations between phylogeny and/or pattern of social arrangements (Rehorek et al. 2010). Although the facial glands of the hoary bat have not been described prior to this study, our results indicate that the overall histological structure of this region is not distinct morphologically from the facial or orbitofacial glands of other Vespertilionid bats studied (Dalquest and Werner 1954; Rehorek et al. 2010). Our histological analysis using compound light microscopy shows that both sebaceous glands and sudoriferous glands contribute to the facial secretions in the hoary bat. Determining the exact nature of the secretory mechanisms, particularly with attention to the large sebaceous glands and the inordinately abundant sudoriferous glands, would best be assessed using transmission electron microscopy. Functionality of the region remains largely unknown, but prior work has identified correlations between this region and the social organization of bat taxa (Rehorek at al. 2010). Haffner (2000) stated that in most Vespertilionid bats (which includes hoary bats) with facial glands, the primary function is grooming. But scent-marking by those with sebum storage capabilities (i.e., more complex glands) can be a secondary function. Using a combination of secretions (including urogenital), female big brown bats (*Eptesicus fuscus*) were capable of recognizing individuals from their maternity colony, as intra-colony members have more similar chemical signatures than extra-colony females (Bloss et al. 2002).

In our study, we report facial gland secretions of a captive adult male hoary bat that occurred October through February, although the samples analyzed on GC-MS were limited to November sampling and the histology from February. While other studies relied on implied function of these glands based on morphology, behavior, phylogeny and/or social arrangements of various taxa, we were able to more directly connect the structure of the actively secreting facial glands with the secretions that they produce during the mating season in an active male signaler. Through our histological analysis, we were able to capture a snapshot of the sudoriferous glands that were actively producing the secretions collected for chemical analysis, and which are likely important to chemical communication among conspecifics.

After methanol extraction the secretion samples were analyzed on GC-MS and found to be complex mixtures consisting of alcohols, alkanes, aldehydes, ketones, terpenoids, androgens, and monoacylglycerols. These families of compounds are seen in secretions of the northern yellow-shouldered bat (*Sturnira parviden*s; Faulkes et al. 2019), Indian flying fox (*Pteropus giganteus*), little golden-mantled flying fox (*Pteropus pumilus*), variable flying fox (*Pteropus hypomelanus*), and Malayan flying fox (*Pteropus vampyrus*; Wood et al. 2005). Secretions from other mammals like Tasmanian short-beaked echidnas (*Tachyglossus aculeatus setosus;* Harris et al. 2012) and bontebok (*Damaliscus pygargus*; Burger et al. 1999) also have similar chemical classifications.

Specifically, mono- and di-terpenoids were identified in the secretions. Previous work has shown terpenoids found in mammals could be due to plants encountered by the mammal, secondary metabolites from diet (Salamon and Davies 1998), or could be produced by the apocrine glands (Burger 2004). Studies have also found terpenoids in mammal secretions with no apparent environmental or dietary interactions (Harris et al. 2012). An analysis of echidna secretions found the diterpenoid methyl dehydroabietate which is also seen in our hoary bat sample (Harris et al. 2012). In addition, they identified kaurene from which kaurenoic acid, found in two of our samples, is derived.

The gland secretion was oily and most likely consisted of triacylglycerides, fatty acids, and squalene (Pannkuk 2013a, b). The methanol extraction used is not ideal for detecting and characterizing these hydrophobic compounds and the large molecular weight of the triacylglycerides could not be seen using GC-MS. As expected, no large lipids were identified. However, two monoacyglycerols were seen, 2-monopalmitin and 1-monostearin. These are likely products of triglyceride hydrolysis and products from the lipids found in the oily secretion. Previous studies have found monoacyglycerols on the epidermis of mammals (Frank 2018). A fatty acid methyl ester (FAME) analysis would have allowed for further details of the lipid identities present in the secretion but was not performed due to limited sample size.

Secretions from bat facial glands have been known to contain smaller chemicals involved in social and reproductive signaling. Of interest in our study is the large number of androgen compounds found in the secretions. Steroids found in previous mammal secretions include cholesterol and desmosterol (Burger et al. 1999; Harris et al. 2012), whereas our study found five androstane derivatives. Specifically, 5β-androstane-3β,17β-diol is an endogenous metabolite of testosterone from the 17β-hydroxyl pathway. Captive male variable flying foxes and little golden-mantled flying foxes exhibit reduced testosterone levels in response to stress (Reeder et al. 2002, 2004). Because most male bats do not exhibit elevated stress levels in association with seasonal mating efforts, testosterone levels do play a role in male grey-headed flying foxes (*Pteropus poliocephalus*) harem maintenance or recovery. Males with higher testosterone levels in their blood plasma were capable of maintaining larger harems than those with lower testosterone levels (Klose et al. 2009). Similarly, during the breeding interval, Malayan flying foxes’ testosterone levels were highest during the establishment of breeding groups (Reeder et al. 2006). The male grey-headed flying fox’s sebaceous neck glands are enlarged during breeding season due to increased androgen production (Martin et al. 1995). This androgen-sensitive gland enlargement occurs in other species during the breeding season, like muskrat (*Ondatra zibethicus*; Zhang et al. 2017). The link between these blood plasma androgen levels to volume and composition of facial gland secretions remains unclear, but the testosterone derivatives found in the secretions could be an indicator of higher testosterone being produced by the bat.

Although most mating efforts are concentrated during fall migrations, hoary bats also have been recorded to mate on their wintering grounds (Cryan et al. 2012). Female hoary bats employ delayed fertilization, and could feasibly mate into the early spring with no impact on parturition date (Shump and Shump 1982). Therefore, despite our collection of secretory samples in fall months (October-November) and delayed histological examination until the bat’s death in February, our collective analyses tell a cohesive story about potential mating activity. Specifically, the active secretions by sudoriferous and sebaceous glands even into winter months could be linked to mating readiness. This pattern likely deviates from cave-hibernating bats, who typically ramp up spermatogenesis for ca. 1–3 mo. of the year, coinciding with fall swarm mating efforts. However, indirect evidence suggests a much longer window of spermatogenesis (4 months) for migratory hoary bats (Drueker 1972), which coincides with a longer mating period. This male bat’s captive lifestyle, maintaining relatively warm ambient temperatures through the winter, would further support the potential for mating, similar to bat species in warmer winter climates with minimal changes in temperature or photoperiod. Findley and Jones (1964) suggest that low-latitude hoary bats may mate year-round in such circumstances.

## Conclusion

Our study is limited due to only having one specimen. However, the six secretions collected temporally allowed for a full chemical profile to be determined instead of a single sampling. Additionally, the histology suggests the secretion production is from both the sebaceous (via holocrine secretion) and sudoriferous (via apocrine or “decapitation” secretion) glands. The increased androgen compounds in addition to the active sudoriferous phase secretions suggest that the facial secretions could be part of the bat mating signaling, and that active glands into the winter months would be a sign of continued mating readiness for this migratory species. This is the first known report of *Lasiurus cinereus* facial gland histology and chemical characterization of facial gland secretions.

## Data Availability

No datasets were generated or analysed during the current study.
